# TLR4 Signaling Is Involved in Brain Vascular Toxicity of PCB153 Bound to Nanoparticles

**DOI:** 10.1371/journal.pone.0063159

**Published:** 2013-05-14

**Authors:** Bei Zhang, Jeong June Choi, Sung Yong Eum, Sylvia Daunert, Michal Toborek

**Affiliations:** 1 Graduate Center for Nutritional Sciences, University of Kentucky, Lexington, Kentucky, United States of America; 2 Department of Biochemistry and Molecular Biology, University of Miami School of Medicine, Miami, Florida, United States of America; Virginia Commonwealth University, United States of America

## Abstract

PCBs bind to environmental particles; however, potential toxicity exhibited by such complexes is not well understood. The aim of the present study is to study the hypothesis that assembling onto nanoparticles can influence the PCB153-induced brain endothelial toxicity via interaction with the toll-like receptor 4 (TLR4). To address this hypothesis, TLR4-deficient and wild type control mice (males, 10 week old) were exposed to PCB153 (5 ng/g body weight) bound to chemically inert silica nanoparticles (PCB153-NPs), PCB153 alone, silica nanoparticles (NPs; diameter, 20 nm), or vehicle. Selected animals were also subjected to 40 min ischemia, followed by a 24 h reperfusion. As compared to exposure to PCB153 alone, treatment with PCB153-NP potentiated the brain infarct volume in control mice. Importantly, this effect was attenuated in TLR4-deficient mice. Similarly, PCB153-NP-induced proinflammatory responses and disruption of tight junction integrity were less pronounced in TLR4-deficient mice as compared to control animals. Additional *in vitro* experiments revealed that TLR4 mediates toxicity of PCB153-NP via recruitment of tumor necrosis factor-associated factor 6 (TRAF6). The results of current study indicate that binding to seemingly inert nanoparticles increase cerebrovascular toxicity of PCBs and suggest that targeting the TLR4/TRAF6 signaling may protect against these effects.

## Introduction

Environmental exposure to polychlorinated biphenyls (PCBs) is an ongoing environmental problem. Because of their chemical stability, slow degradation rate, and high tendency to bioaccumulate in the food chain, PCBs are among the most persistent and widespread organic pollutants [1]. The fate and transport of PCBs are associated with the specific structure of individual PCB congeners. PCBs are readily adsorbed onto particles, such as atmospheric particulates, soil, and sediments. Recent publications have reported PCBs bound to particles ranging from 0.95 µm to 1.5 µm in ambient air [2]–[4].

Exposure to PCBs has been linked to various adverse health effects in humans. Recent reports from a PCB contaminated site located in Anniston, AL indicated that increased serum PCB levels are highly associated with increased prevalence of diabetes [5] and hypertension [6]. These diseases are considered to be risk factors for stroke. In fact, an increase in the incidence of stroke was observed in people exposed to PCBs [7], [8] and living in proximity to PCB hazardous wastes [9]. These observations are important because stroke is one of the leading causes of death worldwide.

A functional blood-brain barrier (BBB) is a the key element for the homeostasis of the central nervous system (CNS). The BBB consists of highly specialized brain endothelial cells which are characterized by the unique phenotype of intercellular tight junctions (TJs) and numerous polarized transport systems [10]. Disruption of TJ proteins is often observed during acute and chronic diseases of the CNS, including stroke [11]. Our research group reported that oral administration of selective PCB congeners resulted in accumulation of PCBs in brain tissue and increased permeability of the BBB [12]–[14]. Highly chlorinated *ortho*-PCBs preferentially accumulate in brain tissue and are associated with several CNS diseases, such as Parkinson’s disease [15] or developmental alterations [16]. The most representative *ortho*-PCB congener is 2,2′,4,4′,5,5′-hexachlorobiphenyl (PCB153), which is commonly detected in human [17], [18] and in environmental samples, including atmospheric particulates [19], [20]. We hypothesize that binding of PCB153 to nanoparticles (NPs) can influence their toxic properties. However, the mechanisms by which PCB153-NP complexes are sensed and transduced via cellular signaling are largely unknown.

Biological systems universally respond to various stimuli of environmental signals by using evolutionarily conserved mechanisms [21], [22]. One such example is toll-like receptors (TLRs), which recognize and respond to an expansive variety of environmental and pathogen associated molecular stimuli [23], [24]. TLRs are widely expressed in various cell types in the brain, including microglia, astrocytes, neurons, and endothelial cells [25], [26]. Recent evidence indicates that TLR4, the first characterized of mammalian TLRs, may play a vital role in ischemia/reperfusion injury [27], [28].

In the present study, we hypothesize that exposure to PCB153 assembled onto nanoparticles contributes to the development of stroke by disruption of the integrity of the cerebral endothelium and induction of proinflammatory responses through stimulation of TLR4 signaling. The results of the present study support this notion and indicate that targeting of the TLR4/tumor necrosis factor-associated factor 6 (TRAF6) signaling can protect against cerebrovascular toxicity of PCB153-NP complexes.

## Materials and Methods

### Materials

2,2′,4,4′,5,5′-hexachlorobiphenyl (PCB153) congener was purchased from AccuStandard (New Haven, CT) and silica NPs from NanoAmor (Houston, TX). Characterization of NPs and construction of silica NPs coated with PCB153 were described in our previous study [29]. Briefly, silica NPs (80 mg) and PCB153 (10 mg) were dispersed in acetone and sonicated to prevent aggregation. The highly hydrophobic surface character of PCB153 and silica NPs allows them to interact with each other based on electrostatic attraction. After evaporation of acetone, the particles were resuspended in phosphate buffered saline (PBS) or cell culture medium, sonicated, and centrifuged at 12,000 rpm for 5 min. The supernatant containing PCB153-NPs was then collected to analyze PCB153 levels by gas chromatography/mass spectrometry (GC/MS). The hydrodynamic size distribution and the amounts of PCB153-NPs were monitored by dynamic light scattering (DLS) and atomic force microscope (AFM), respectively. Control NPs were prepared using the same procedure, without adding PCB153. All treatment factors were tested for possible endotoxin contamination using the LAL chromogenic endotoxin quantitation kit (Thermo Scientific Pierce, Rockford, IL). The levels of endotoxin in all preparations were below the detection limit, indicating no contamination.

### Experimental Groups and Surgical Procedures

All experimental procedures and protocols were approved by the National Institutes of Health Guide for the Care and Use of Laboratory Animals. C3H/HeJ mice contain a point mutation in the TLR4 gene and are TLR4-deficient, whereas C3H/HeouJ mice express normal TLR4 activity and were used as controls. Mice (males, 10–12 weeks old; Jackson Laboratories) were infused with a) PCB153 bound to nanoparticles (PCB153-NPs), b) PCB153 dissolved in 0.01% DMSO (PCB153), c) nanoparticles (NPs) alone, or d) vehicle (PBS). PCB153 was administered in the amount of 5 ng/g body weight. All infusions were performed through the internal carotid artery (ICA) using a surgical technique standardized by our research group [30] for selective drug delivery into the brain vasculature.

Transient focal cerebral ischemia was induced by a 40 min occlusion of the middle cerebral artery (MCA), following a 24 h reperfusion as described previously [31]. This procedure is frequently used to induce experimental stroke model. During standardization, we established that a 40 min MCA occlusion was relatively well-tolerated and did not induce mortality as assessed 24 h after the procedure. Briefly, in anesthetized mice, a 6–0 surgical nylon suture coated with silicon (Doccol, Redlands, CA) was advanced through the left common carotid artery and up to the ICA to block the origin of the MCA. After occlusion for 40 min, reperfusion was initiated by removing the suture to restore the blood flow.

### Assessment of the Infarct Volume

The mouse brain was removed and sectioned into 7 coronal slices with 1 mm thickness from the frontal pole to the occipital pole using a coronal acrylic matrice (Braintree Sci., Braintree, MA). The brain slices were then stained with 2% 2,3,5-triphenyltetrazolium chloride (TTC) at 37°C for 20 min. The viable brain tissue was stained in red, whereas the infarcted area appeared unstained. The infarct size and volume were calculated using ImageJ software as previously described [32].

### Brain Microvessel Isolation

Isolation of brain microvessels was performed as described previously [14]. After removing meninges and choroids plexus, brain tissue was homogenized in ice-cold buffer containing 103 mM NaCl, 4.7 mM KCl, 2.5 mM CaCl_2_, 1.2 mM KH_2_PO_4_, 1.2 mM MgSO_4_, 15 mM HEPES, 25 mM NaHCO_3_, 10 mM glucose, 1 mM Na pyruvate, 10 g/L dextran and protease inhibitor cocktail tablets (Roche Diagnostics, Indianapolis, IN). The homogenates were mixed with 26% dextran and centrifuged at 5,800× g at 4°C for 20 min. The collected pellets were resuspended in ice-cold buffer and filtered through a 70 µm cell strainer (BD Biosciences, San Jose, CA). Filtered samples were re-pelleted by centrifugation, followed by either resuspension in 150 µL of 6 M urea lysis buffer for Western blot analyses, or resuspension in 200 µl of TRIZOL (Invitrogen, Carlsbad, CA) for total RNA extraction.

### Cell Cultures, Treatment Factors, and Gene Silencing

Human brain endothelial cells (hCMEC/D3 cell line) were developed by Weksler et al. [33]. They represent a stable, well characterized, and differentiated cell line. Cells were cultured as previously described [34]. Confluent cultures were exposed to PCB153-NPs, NPs, PCB153 alone, or vehicle for 24 h. In cell culture experiments, PCB153 was used in subtoxic concentration of 1.6 μM, which is lower than the levels reported in humans acutely exposed to PCBs [35], [36]. In selected experiments, cultured cells were treated with 10 μM CLI095, a pharmacological inhibitor of TLR4, which blocks the signaling mediated by the intracellular domain of TLR4.

Cultured cells at 70–80% confluency were transfected with 60 nM of control or TRAF6 specific siRNA (Applied Biosystems, Carlsbad, CA) using GeneSilencer (Genlantis, San Diego, CA). The cells were incubated with transfection mixtures for 24 h and allowed to recover in complete medium for 48 h before exposure to PCB153 and/or NPs.

### Immunoblotting and Immunoprecipitation

Immunoblotting was performed with either whole cell lysates (30 µg protein per sample) prepared in RIPA lysis buffer (50 mM Tris–HCl pH 7.4, 1% NP-40, 0.25% sodium deoxycholate, 150 mM NaCl, and 1 mM EDTA) or lysed mouse brain microvessels (50 µg protein per sample). Protein samples were separated on SDS-polyacrylamide gel, blotted onto polyvinyl difluoride membranes (Bio-Rad Laboratories, Hercules, CA), and incubated with the respective antibodies. Anti-occludin and anti-claudin-5 antibodies were from Invitrogen, anti-TLR4 antibody from Santa Cruz Biotechnology (Santa Cruz, CA), anti-actin antibody from Sigma, and all secondary antibodies from Cell Signaling Technology (Danvers, MA). For visualization of detected proteins, immunoblots were analyzed using an ECL Western blot detection kit (GE Healthcare Life Sciences, Piscataway, NJ) and proteins of interest were semi-quantitated with ImageJ software.

Immunoprecipitation of TRAF6 was performed using 800 µg of protein extracted from whole cell lysate. Samples were incubated with 1 μg of anti-TRAF6 antibody (Cell Signaling Technology) overnight at 4°C. Next day, 30 μL of Protein A/G Plus Agarose (Thermo Scientific Pierce, Rockford, IL) was added to each sample and immunoprecipitation was performed for 2 h at 4°C. Bound proteins were eluted by boiling in SDS sample buffer for 5 min and analyzed on SDS-polyacrylamide gel.

### Real-time RT-PCR

Total RNA was extracted from freshly isolated microvessels using TRIZOL reagent (Invitrogen) according to the manufacturer’s instructions with an additional chloroform extraction, phase separation, and an extra wash in 70% ethanol. Then, 1 µg of RNA was reverse-transcribed using the Reverse Transcription System (Promega, Madison, WI) and 3 µL of final RT product was used for PCR amplification. Taqman Universal PCR Master Mix, pre-developed primer pairs and probes were purchased from Applied Biosystesms (Foster City, CA). The following thermocycling conditions were employed: 95°C for 10 min, followed by 95°C for 15 sec, and 60°C for 60 sec (for up to 40 cycles). Expression of mRNA was calculated and analyzed by the comparative C_T_ method as described [37].

### ELISA

The levels of cytokines and chemokines in cell culture media were determined using Multi-Analyte ELISArray kit (Qiagen, Valencia, CA). Briefly, 50 μL aliquots of culture media were added into individual wells of the ELISArray kit and incubated at room temperature for 2 h. After the plate was washed three times with washing buffer, 50 μL of biotin-conjugated anti-IL-6, anti- CXCL-8, anti-CCL-2, and anti-CCL-5 antibodies were added into indicated wells and incubated at temperature for 1 h. Then, the plate was washed three times and avidin-conjugated horseradish peroxidase was added to each well for 30 min incubation at room temperature, followed by four washings with the washing buffer. After 15 min incubation with development solution, stop solution was added to each well and the absorbance was measured at 450 nm using SpectraMax 190 absorbance microplate reader (Molecular Devices, Sunnyvale, CA). The standard curve was generated using antigen standard of each target protein at the concentrations between 0 to 200 pg/mL.

### Statistical Analysis

Statistical analysis was completed using SigmaPlot 12.0 (Systat Software, San Jose, CA). One-way or Two-way ANOVA followed by Holm-Sidak *post hoc* test was used to compare mean responses among the treatments. A statistical probability of *p*<0.05 was considered significant.

## Results

### TLR4 Deficiency Diminishes PCB153-NP-induced Enhancement of Infarct Volume and Disruption of the BBB Integrity

**Figure 1:**
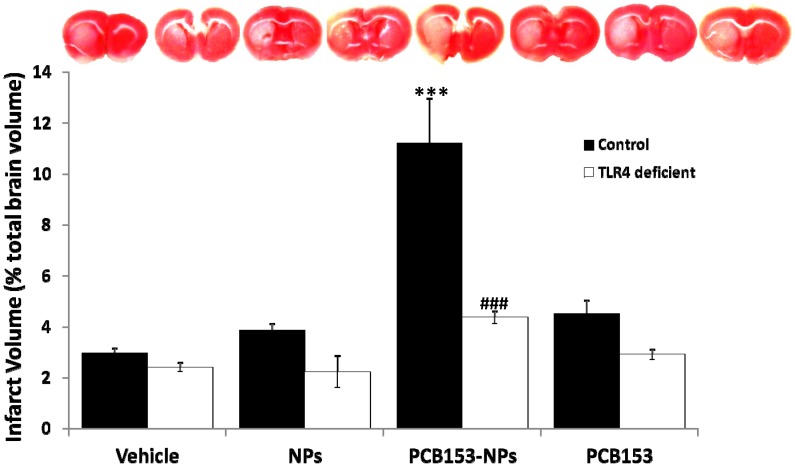
PCB153-NP-induced enhancement of infarct volume following ischemia/reperfusion is reduced in TLR4-deficient mice. Mice were exposed to PCB153-NPs (5 ng PCB153/g body weight bound to 1.04×10^5^ silica NPs) by infusion into the internal carotid artery (ICA). Control mice were infused with the same amounts of NPs, PCB153 or vehicle (PBS). After 24 h, all animals were subjected to a 40 min occlusion of the middle cerebral artery (MCA), followed by a 24 h reperfusion. The infarct area was detected by 2,3,5-triphenyltetrazolium chloride (TTC) staining and the image illustrates the representative results. The loss of tissue viability is reflected by unstained (white) areas. Quantified results are depicted in a bar graph. Results are means ± SEM, n = 5. Significantly different as compared to vehicle treatment in mice with normal TLR4 expression at ***p<0.001. Results in the TLR4-deficient mice treated with PCB153-NPs are statistically different from those in control animals exposed to PCB153-NPs at ^###^p<0.001.

To test the hypothesis that PCB153-NPs potentiate the ischemic injury through activation of TLR4, mice with a point mutation in the TLR4 gene (C3H/HeJ) and mice expressing normal TLR4 activity (C3H/HeouJ) were employed. As indicated in Figure 1, exposure to PCB153-NPs significantly increased the infarct volume in the control mice as compared to treatment with vehicle (PBS), NPs, or PCB153 alone. However, TLR4-deficient mice infused with PCB153-NPs showed significantly smaller infarct volume as compared to control mice.

**Figure 2:**
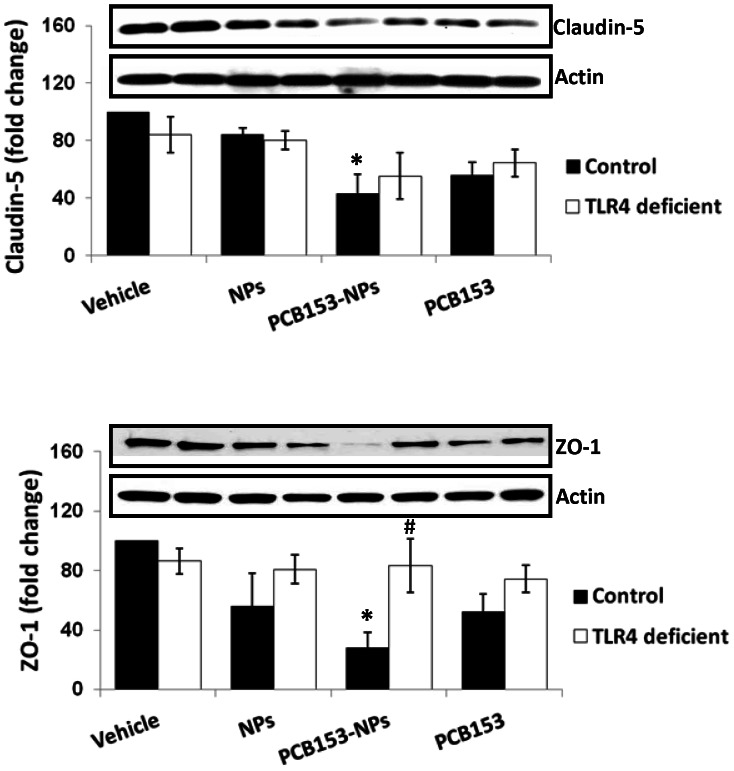
TLR4 deficiency protects against PCB153-NP-induced TJ protein disruption. Mice were infused with PCB153-NPs or control treatments as in Figure 1. Expression of ZO-1 and claudin-5 was analyzed in brain microvessels isolated from TLR4-deficient or control mice by immunoblotting. Results are means ± SEM, n = 5. Significantly different as compared to control treatments in normal mice at *p<0.05. Results in the TLR4-deficient mice treated with PCB153-NPs are statistically different from those in control animals exposed to PCB153-NPs at ^#^p<0.05.

**Figure 3:**
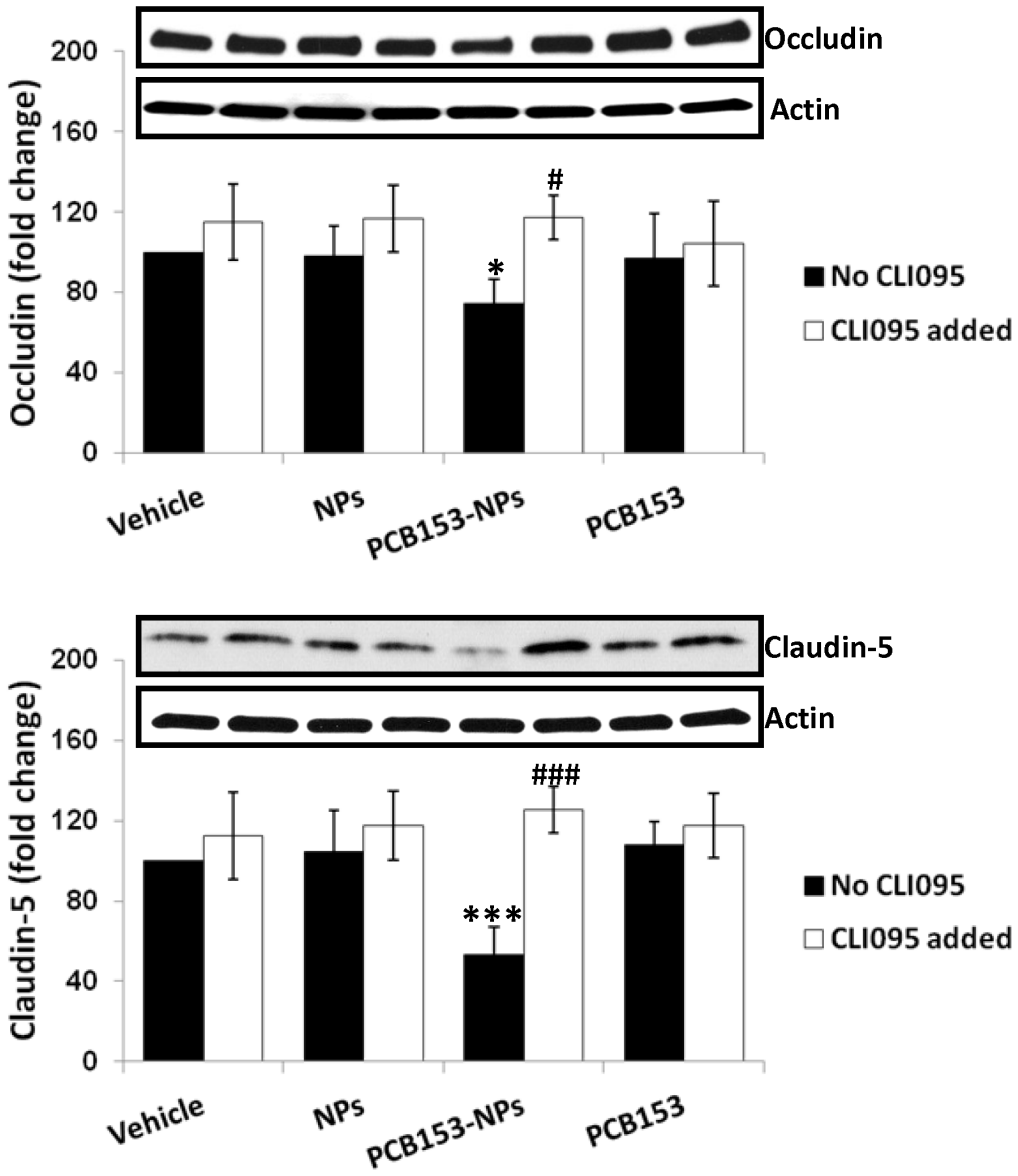
Inhibition of TLR4 prevents PCB153-NP-induced TJ protein disruption in human brain endothelial cells. Conuent brain endothelial cell cultures were treated with PCB153-NPs (PCB153, 1.6 µM; NPs, 2.08×10^5^), or the same amounts of PCB153, NPs, or vehicle for 24 h. Selective cultures were pretreated with TLR4 inhibitor CLI095 (10 μM) or vehicle (DMSO, 0.01%) for 1 h, followed by co-exposure to PCB153 and/or NPs for 24 h. TLR4 inhibitor was retained in media throughout PCB153 and/or NPs treatment. Occludin and claudin-5 levels were measured by immunoblotting. Results are means ± SD, n = 5. Significantly different as compared to vehicle at *p<0.05 or ***p<0.001. Results in cultures pretreated with CLI095 are statistically different from those in the corresponding cultures without added CLI095 at ^#^p<0.05 or ^###^p<0.001.

Disruption of TJs is a typical event during cerebral ischemia. Therefore, we evaluated the effects of PCB153 and/or NPs on expression of transmembrane TJ proteins, such as occludin and claudin-5 as well as TJ-associated protein ZO-1 both in animals and cell cultures of brain endothelial cells. Exposure to PCB153-NPs but not to PCB153 or NPs alone resulted in a decrease in claudin-5 and ZO-1 levels in mice with normal TLR4 expression, whereas deficiency of TLR4 diminished the effect (Figure 2). Consistent with these results, levels of occludin and claudin-5 were also markedly reduced following exposure to PCB153-NPs in brain endothelial cells. Importantly, inhibition of TLR4 activity with CLI095 attenuated these effects (Figure 3), further indicating that TLR4 pathways is involved in PCB153-NP-induced alteration of TJ expression.

**Figure 4:**
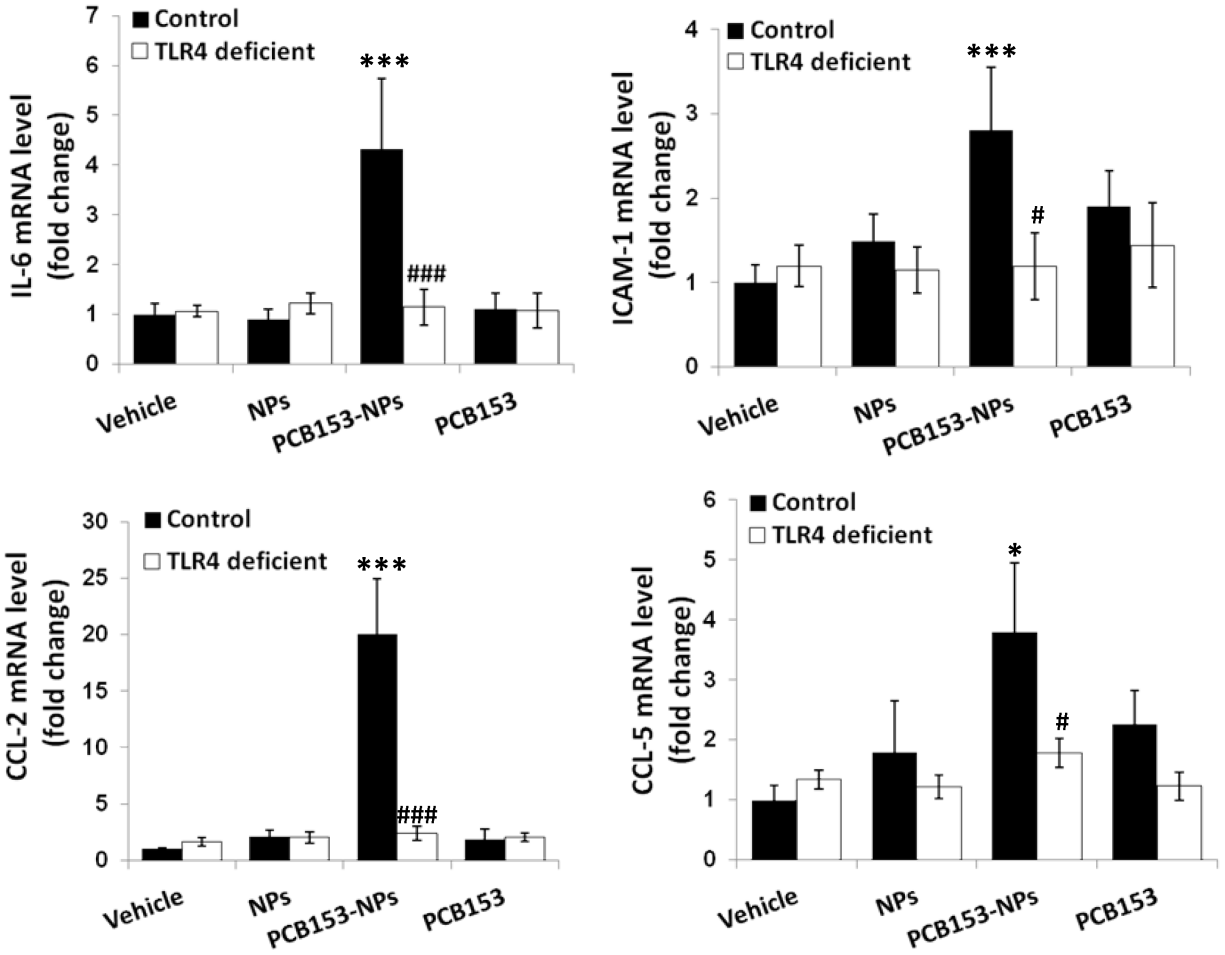
TLR4 deficiency protects against PCB153-NP-induced expression of proinflammatory genes in brain microvessels. Mice were treated as in Figure 1. mRNA levels of IL-6, ICAM-1, CCL-2 (MCP-1) and CCL-5 (RANTES) were determined in isolated brain microvessels by real-time PCR. Results are means ± SEM, n = 5. Significantly different as compared to vehicle treatments in normal mice at *p<0.05, or ***p<0.001. Results in the TLR4-deficient mice treated with PCB153-NPs are statistically different from those in control animals exposed to PCB153-NPs at ^#^p<0.05 or ^###^p<0.001.

**Figure 5:**
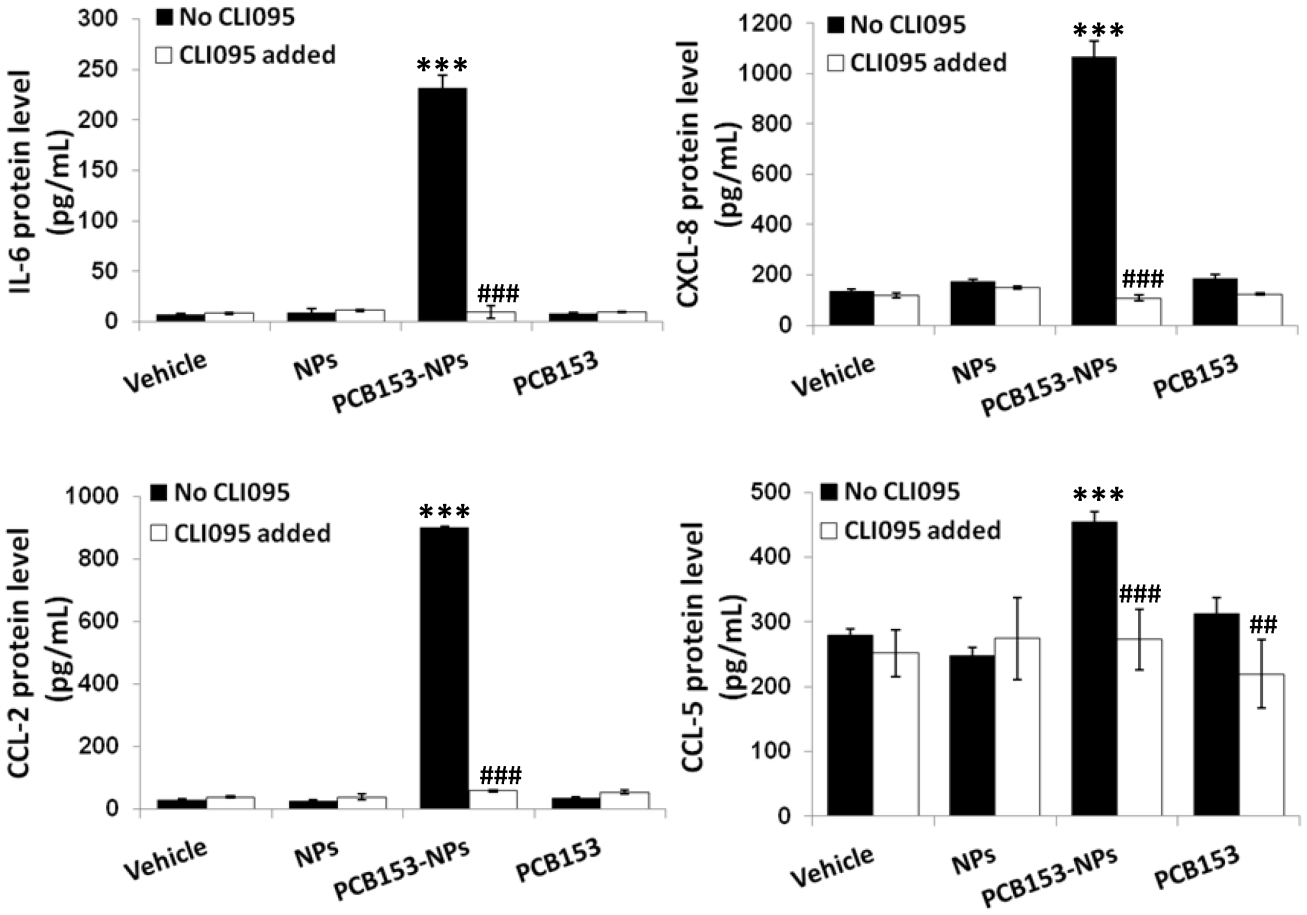
Inhibition of TLR4 prevents PCB153-NP-induced proinflammatory responses in human brain endothelial cells. Confluent brain endothelial cells were treated as in Figure 3 for 24 h. Protein levels of IL-6, CXCL-8 (IL-8), CCL-2 and CCL-5 were determined by ELISA in cell culture supernatants. Significantly different as compared to vehicle at ***p<0.001. Results in cultures pretreated with CLI095 are statistically different from those in the corresponding cultures without added CLI095 at ^##^p<0.01 or ^###^p<0.001.

Overexpression of proinflammatory cytokines, chemokines, and adhesion molecules in the brain is hallmark of neuroinflammation [38]. Therefore, we evaluated the expression levels of proinflammatory cytokines (IL-6 and CXCL-8 [IL-8]), chemokines (CCL-2 and CCL-5 [RANTES]) and adhesion molecule ICAM-1 following exposure to PCB153 and/or NPs in brain microvessels and cultured brain endothelial cells. Working in concert, these proinflammatory mediators target the subsequent critical steps of neuroinflammatory responses, such as inflammatory cell attraction to the proximity of the endothelium, adhesion, and transendothelial migration. As shown in Figure 4, mRNA levels of IL-6, CCL-2, CCL-5, and ICAM-1 were significantly elevated in brain capillaries of wild type mice exposed to PCB153-NPs but not to PCB153 or NPs alone. Importantly, deficiency of TLR4 effectively protected against these effects. Inhibition of TLR4 signaling by CLI095 also attenuated PCB153-NP-induced overproduction of IL-6, CXCL-8, CCL-2 and CCL-5 protein levels in cultured human brain endothelial cells (Figure 5).

### Exposure to PCB153-NPs Induces TRAF6 Interaction with TLR4

**Figure 6:**
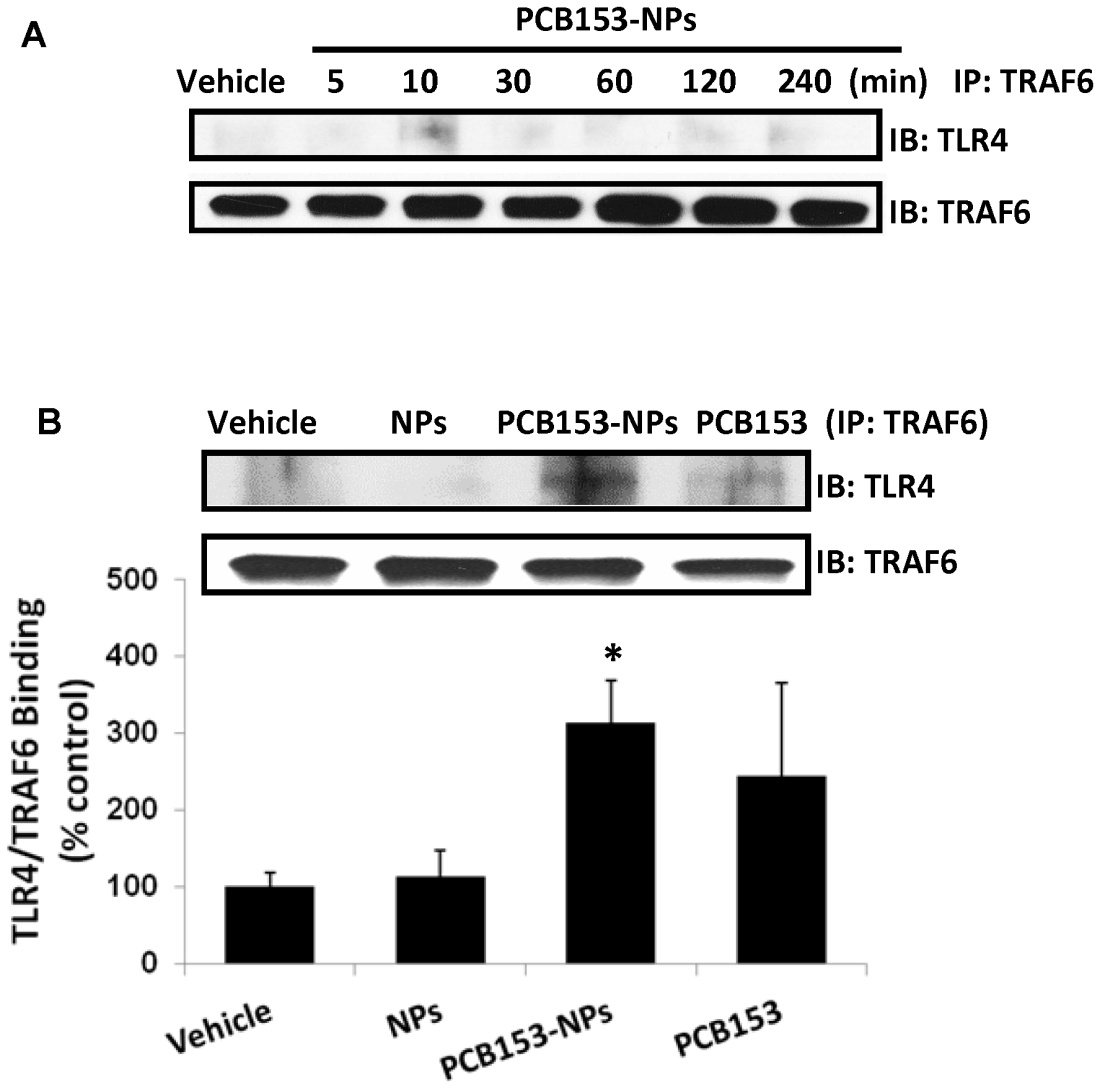
Exposure to PCB153-NPs induces TLR4 interaction with TRAF6. (A) Confluent brain endothelial cells were treated with PCB153-NPs for the indicated time. Cellular extracts were immunoprecipitated using anti-TRAF6 antibody, followed by immunoblotting with anti-TLR4 antibody. (B) Confluent brain endothelial cells were exposed to PCB153 and/or NPs for 10 min, followed by determination of TLR4 interaction with TRAF6 as in (A). The blot illustrates the representative data of four independent experiments and the bar graph shows quantified results. Results are the mean ± SD, n = 4. Significantly different as compared to vehicle treatments in normal mice at *p<0.05.

Upon activation, TLRs recruit adaptor molecules, such as MyD88, which then activate a series of downstream signaling molecules, including TRAF6 [24]. To investigate these events, brain endothelial cells were treated with PCB153-NPs for up to 4 h. Cell lysates were then immunoprecipitated with anti-TRAF6 antibody and probed for TLR4. Figure 6A indicates that PCB153-NPs induced a rapid (10 min) but transient recruitment of TRAF6 to TLR4. Treatment with PCB153 alone for 10 min also resulted in binding of TRAF6 to TLR4; however, this effect was less prominent as compared to PCB153-NPs (Figure 6B).

### TRAF6 Mediates PCB153-NP-induced Alterations in TJ Protein Expression and Proinflammatory Responses

**Figure 7:**
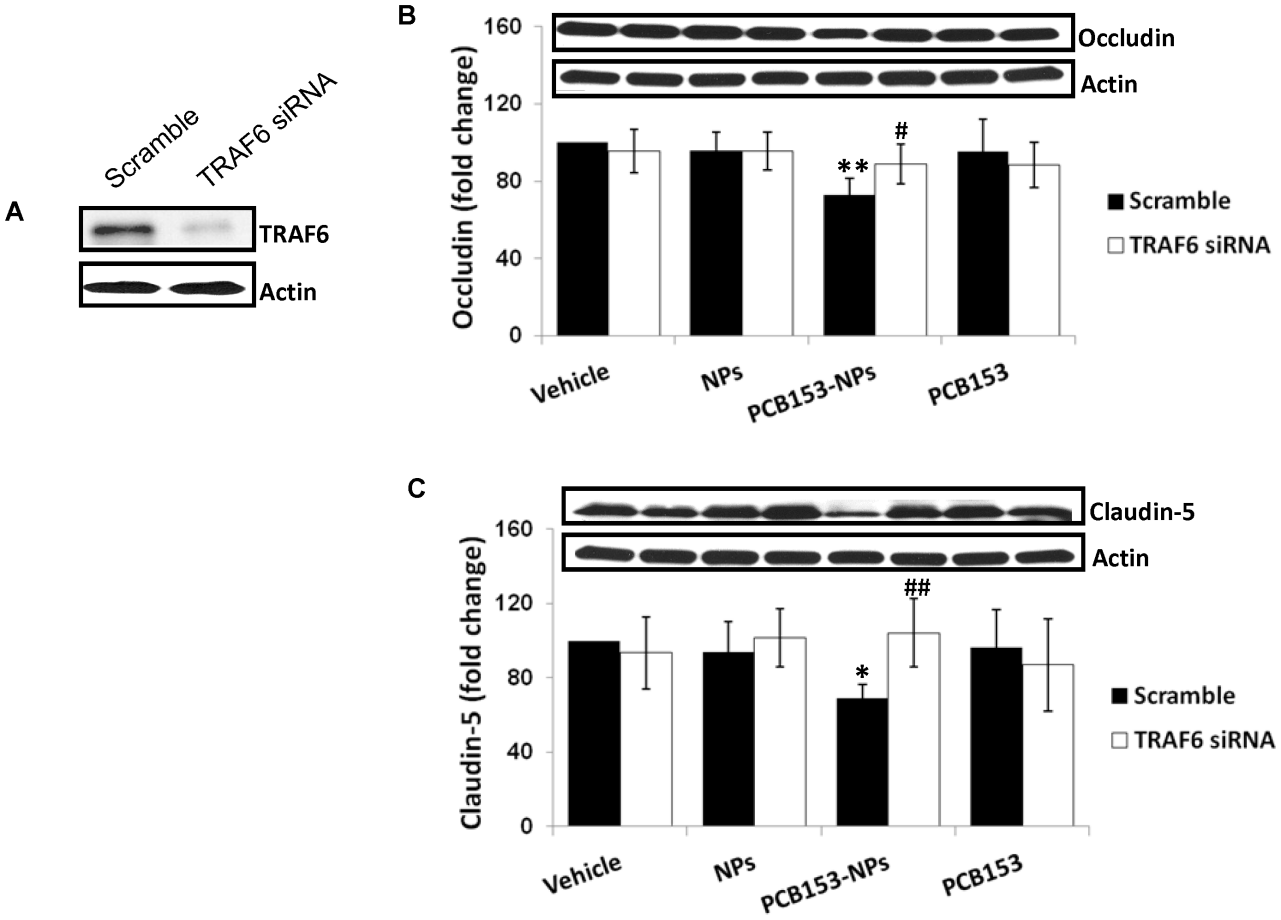
TRAF6 mediates PCB153-NP-induced a decrease in TJ protein expression. (A) Immunoblotting illustrating the efficiency of TRAF6 silencing. (B and C) Human brain endothelial cells were transfected with TRAF6 siRNA, followed by treatment with PCB153 and/or NPs as in Figure 3 for 24 h. Expression of occludin (B) and claudin-5 (C) was assessed by immunoblotting. The blot illustrates the representative data of four independent experiments and the bar graph shows quantified results. Results are means ± SEM. Significantly different as compared to vehicle at *p<0.05 or **p<0.01. Results in cultures with silenced TRAF6 are statistically different from those in the corresponding cultures transfected with control (scrambled) siRNA at ^#^p<0.05 or ^##^p<0.01.

To investigate the involvement of TRAF6 in PCB153-NP-mediated TJ disruption, expression of TRAF6 in brain endothelial cells was silenced with TRAF6 siRNA (Figure 7A), followed by exposure to PCB153 and/or NPs for 24 h. The results indicated that silencing of TRAF6 markedly protected against PCB153-NP-mediated reduction in occludin (Figure 7B) and claudin-5 (Figure 7C) protein levels.

**Figure 8:**
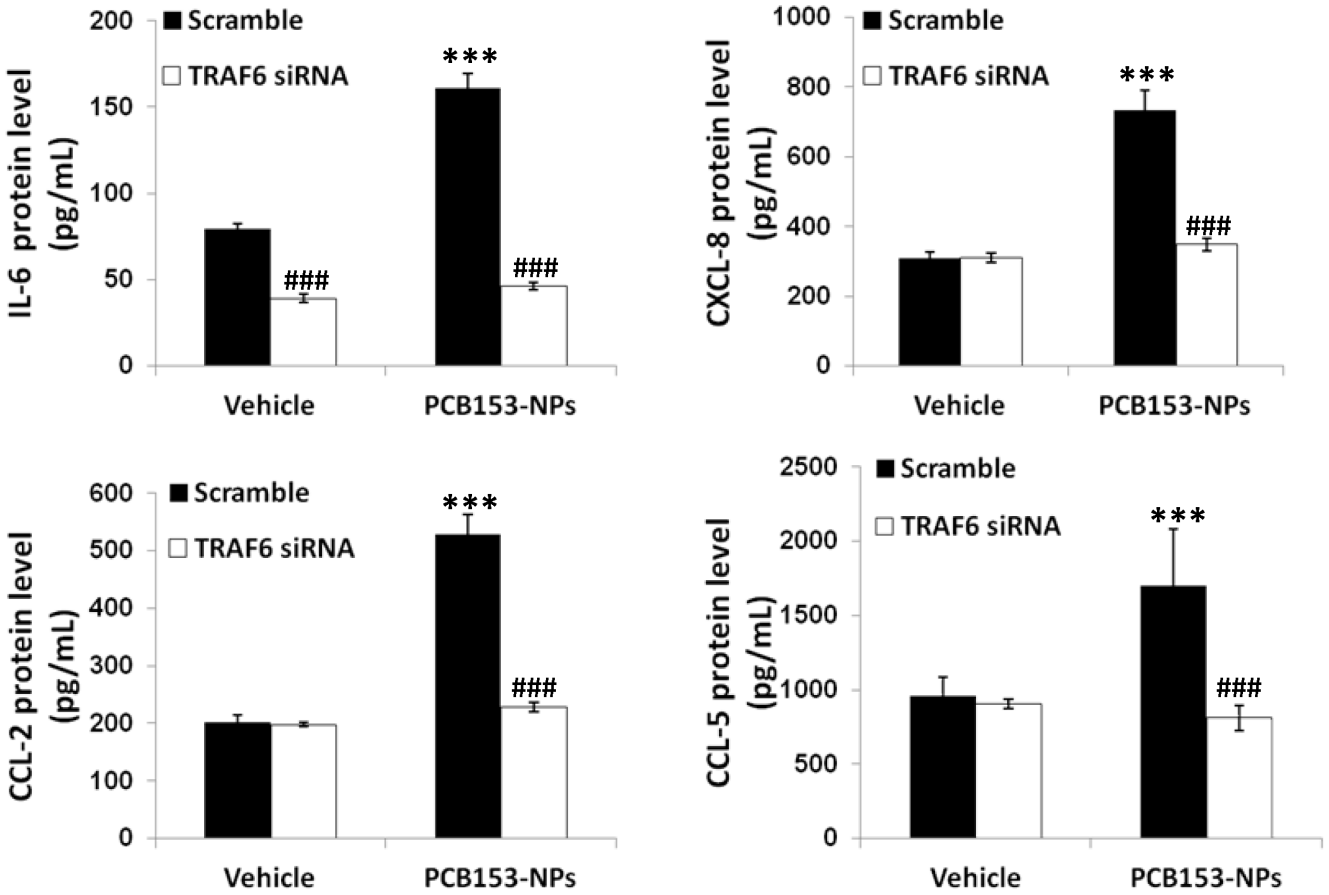
TRAF6 mediates PCB153-NP-induced production of inflammatory mediators. Human brain endothelial cells were treated as in Figures 7B and C, followed by the assessment of IL-6, CXCL-8, CCL-2 and CCL-5 in culture medium by ELISA. Results are means ± SD, n = 4. Significantly different as compared to vehicle at ***p<0.001. Results in cultures with silenced TRAF6 are statistically different from those in the corresponding cultures transfected with control (scrambled) siRNA at ^###^p<0.001.

In the last series of experiments, we investigated the role of TRAF6 in PCB153-NP-stimulated production of inflammatory mediators. Consistent with the results in Figure 5, exposure to PCB153-NPs significantly increased the production of IL-6, CXCL-8, CCL-2 and CCL-5 in brain endothelial cells transfected with scrambled (control) siRNA. Importantly, silencing of TRAF6 effectively reduced the production of these inflammatory mediators in response to PCB153-NPs (Figure 8).

## Discussion

While the cellular effects of dioxin-like PCBs are linked to activation of the aryl hydrocarbon receptor (AhR), signal transduction mechanisms induced by *ortho*-PCBs are complex and include more diverse number of receptors and signaling pathways. Non-coplanar PCBs, such as PCB153 used in the present study, possess at least two *ortho* chlorines on the biphenyl ring, which generate steric forces that rotate the ring structure away from a single plane. Such a structure precludes interactions with the AhR; however, *ortho*-PCB congeners can act as ligands for the constitutive andorstane receptor (CAR) and/or the pregnane-X receptor (PXR), and activate genes targeted by these receptors [39]. In addition to the nuclear receptors, ryanodine receptors (RyRs) have also been identified as candidates to mediate *ortho*-PCB-induced perturbations in cellular Ca^2+^ signaling, which plays a pivotal role in metabolism, proliferation, gene transcription, and protein translation in almost all cell types [40]. For example, PCB95 and PCB153 at concentrations lower than 1 μM were shown to significantly enhance activity of RyR1 and RyR2 [41]. Furthermore, *ortho*-PCBs can activate several signaling cascades including Janus kinase (JAK), epidermal growth factor receptor (EGFR), Src kinase, and mitogen-activated protein kinase (MAPK) [42]–[44]. We demonstrated that PCB153 upregulates expression of ICAM-1 and VCAM-1 through the Src/JAK/EGFR redox signaling, which is triggered by the NADPH oxidase-mediated increase of superoxide generation [34].

In the current study, we present evidence that TLR4 is yet another cellular receptor that is involved in *ortho*-PCB-mediated vascular toxicity [45]. While it is generally accepted that TLRs are sensors of a variety of biological molecules, like polysaccharides, proteins and nucleic acids [46], our observations that a non-biological material, such as PCB153-NPs, can act via the TLR4 signaling pathway are novel. By inhibition of TLR4 activity via pharmacological inhibitors and by using TLR4 deficient mice, we demonstrated that proinflammatory effects of PCB153-NPs are sensed via TLR4 in both brain microvessels and brain endothelial cells. These proinflammatory mediators are actively involved in the development of cerebrovascular and neurovascular alterations. ICAM-1 is an adhesion molecule which stimulates firm adhesion of leukocytes to the vascular endothelium and plays a critical role in the pathology of numerous proinflammatory vascular diseases, including atherosclerosis [47]. CXCL-8 is one of the CXC chemokine members that has potent chemotactic activity for neutrophils [48]. It has also been shown that CXCL-8 can induce generation of superoxide and hydrogen peroxide [49] as well as increase expression of adhesion molecules [50], [51]. CC chemokines, such as CCL-2 and CCL-5, are implicated in the activation of monocytes, macrophages and lymphocytes [52]. Additionally, CCL-2 stimulates monocytes to produce tissue factor and proinflammatory cytokines, including IL-6 [36]. An elevated IL-6 level is associated with an increased infarct volume and severity of stroke outcome [53], [54].

Activation of TLR4 results in interaction of its intracellular TIR domain with MyD88, whose amino-terminal death domain (DD) associates with the serine kinase IL-1 receptor-associated kinase (IRAK). These events subsequently recruit TRAF6 [23], followed by nuclear translocation of proinflammatory transcription factors NF-κB and AP-1. In agreement with this general pathway, we observed that treatment of brain endothelial cells with PCB153-NPs resulted in binding of TRAF6 to TLR4. While these interactions were transient, their importance was evident as silencing of TRAF6 significantly attenuated PCB153-NP-induced overproduction of inflammatory mediators.

Although the involvement of TLR4 in modulating BBB disruption has been reported [55], the precise mechanisms involved are not fully understood. Therefore, our observation that TLR4 signaling modulates PCB153-NP-induced disruption of TJ protein expression is another novel finding in the current study. We propose that TLR4-mediated an increase in inflammatory mediators may be responsible, at least in part, for these effects. Indeed, CCL-2 has been reported to induce occludin phosphorylation on both serine/threonine residues, resulting in increased BBB permeability. Furthermore, CCL-2 targets ZO-1 and claudin-5 phosphorylation through a signaling pathway involving Rho and protein kinase C (PKC) [56], [57]. Evidence for the phosphorylation and ubiquitin-mediated proteasomal degradation of TJ proteins has been demonstrated previously [58], [59]. In addition, TLR4/TRAF6 signaling can stimulate activation of matrix metalloproteinase-9 (MMP-9) [60], an enzyme which is responsible for proteolytic degradation of TJ proteins [61]. It was also reported that TLR4/TRAF6 signaling is involved in nanomaterial-induced autophagy formation [62]. While autophagy is a highly conserved pathway of intracellular protein degradation [63], [64], our laboratory provided evidence that stimulation of autophagy in brain endothelial cells is associated with decreased expression of the TJ proteins [65].

Dysregulation of TLR4 signaling appears to be involved in several disorders, including cerebral ischemia and stroke [28], [66]. Consistent with these reports, we observed that the infarct volume in TLR4-deficient mice treated with PCB153-NPs was significantly decreased as compared to mice with normal expression TLR4. While a variety of factors can contribute to the development of stroke, the pathology of ischemia/reperfusion has a very strong inflammatory component [67]. Therefore, inflammatory responses induced by PCB153-NPs in cerebral vessels are likely to be responsible for the development of enhanced brain infarct. BBB breakdown, due to disruption of TJs and infiltration with inflammatory cells, may be another contributing factor to the progress of the brain injury following ischemia/reperfusion and exposure to PCB153-NPs.

**Figure 9:**
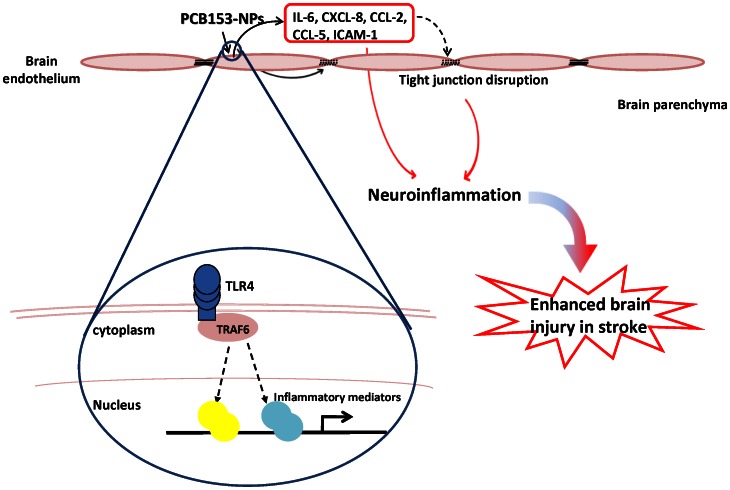
Schematic diagram of PCB153-NP-induced cerebrovascular toxicity via stimulation of the TLR4. Binding of PCB153 to silica nanoparticles activates the TLR4 and triggers its interaction with downstream molecule TRAF6. These events subsequently initiate overexpression of proinflammatory mediated and decreased expression of TJ proteins in brain endothelial cells. Disrupted BBB and enhanced neuroinflammatory responses contribute to enhanced brain injury in experimental stroke model.

In summary, our study demonstrates that exposure to PCB153 bound onto silica nanoparticles triggers TLR4/TRAF6-regulated inflammatory responses and alterations of TJ protein expression, which then contribute to enhanced brain injury following ischemia/reperfusion (Figure 9). These results indicate an important role for TLR4 signaling in PCB-mediated cerebrovascular toxicity, suggesting that this signaling pathway may be a potential target for therapeutic intervention in cerebrovascular disorders.
