# Correction: TLR4 Signaling Is Involved in Brain Vascular Toxicity of PCB153 Bound to Nanoparticles

**DOI:** 10.1371/annotation/e34adc2a-f73a-4055-8519-fa2deb2e852f

**Published:** 2014-01-09

**Authors:** Bei Zhang, Jeong June Choi, Sung Yong Eum, Sylvia Daunert, Michal Toborek

This article was republished on January 8th, 2014. The image for the vehicle control in Figure 1 is an image that originates from the authors’ previous publication in J Neuroimmune Pharmacol., 7:991-1001. This image was used in error in the current article and is subject to copyright, as a result, we are replacing the original Figure 1 by a corrected figure that includes the correct vehicle control.

In addition, there was an error in the preparation of Figure 7. The representative images of occludin and claudin-5 expression (Figures 7B and 7C, respectively) originate from the same sample preparation and from the same membrane. Actin levels were also determined in the same sample preparation; therefore, the same actin image was used or both Figures 7B and 7C. Unfortunately, the film was accidentally flipped when the actin image was scanned for Figure 7C. The authors are making the raw blots for Figure 7B and 7C available with this Correction.

Actin: http://www.plosone.org/corrections/pone.0063159.cn.actin.tif

Occludin: http://www.plosone.org/corrections/pone.0063159.cn.occludin.tif

Claudin-5: http://www.plosone.org/corrections/pone.0063159.cn.claudin5.tif

These mistakes do not affect the results reported in the article.

